# Eight-hour time-restricted feeding improves endocrine and metabolic profiles in women with anovulatory polycystic ovary syndrome

**DOI:** 10.1186/s12967-021-02817-2

**Published:** 2021-04-13

**Authors:** Chunzhu Li, Chuan Xing, Jiaqi Zhang, Han Zhao, Wenjing Shi, Bing He

**Affiliations:** Shengjing Hospital of China Medical University, No. 36 Sanhao Street, Heping District, Shenyang, Liaoning China

**Keywords:** Intermittent fasting, Time-restricted feeding, Polycystic ovary syndrome, Weight-loss, Insulin resistance, Hyperandrogenemia, Anovulation

## Abstract

**Background:**

Time-restricted feeding (TRF) is a form of intermittent fasting, which is beneficial for weight loss and cardiometabolic health. Polycystic ovary syndrome (PCOS) is one of the most common reproductive endocrine and metabolic diseases affecting women of childbearing age. It is associated with an increased prevalence of metabolic syndrome, cardiovascular diseases and type 2 diabetes. The effects of TRF on PCOS patients remains undefined, here we investigated the impact of TRF on women with anovulatory PCOS.

**Methods:**

Eighteen PCOS women aged between 18 and 31 with anovulation participated in a 6-week trial which were divided into two consecutive periods: (1) 1-week baseline weight stabilization period and (2) 5-week TRF period. Fifteen participants completed the study. Changes in body weight, body mass index (BMI), Waist-to-Hip Ratio, skeletal muscle mass, body fat mass (BFM), body fat percentage (BF%), visceral fat area (VFA), luteinizing hormone (LH), follicle-stimulating hormone (FSH), LH/FSH, total testosterone (TT), sex hormone-binding globulin (SHBG), free androgen index (FAI), fasting glucose, fasting insulin (FINS), homeostasis model assessment-insulin resistance (HOMA-IR), area under the curve (AUC) for insulin (AUCIns), area under the curve (AUC) for glucose (AUCGlu), AUCIns/AUCGlu Ratio, lipids, uric acid, alanine aminotransferase (ALT), aspartate aminotransferase, high-sensitivity C-reactive protein (hsCRP), insulin-like growth factor (IGF-1), menstrual cycle and eating behaviors were evaluated.

**Results:**

Significant changes in body weight, BMI, BFM, BF%, VFA, TT, SHBG, FAI, FINS, HOMA-IR, AUCIns, AUCIns/AUCGlu Ratio, ALT, hsCRP and IGF-1 were found after the TRF period. An improvement in menstrual cycle irregularity was detected in 73.3% (11/15) patients.

**Conclusion:**

The diet of TRF may be beneficial to anovulatory PCOS on weight loss especially reducing body fat, improving menstruation, hyperandrogenemia, insulin resistance and chronic inflammation**.**

*Trial registration* Clinicaltrial.gov, NCT04580433, registered October 8, 2020, https://clinicaltrials.gov/ct2/show/NCT04580433.

**Supplementary Information:**

The online version contains supplementary material available at 10.1186/s12967-021-02817-2.

## Background

Polycystic ovary syndrome (PCOS) is one of the most common reproductive endocrine and metabolic disorders that affects up to 10% women of childbearing age [1]. It shows a broad range of reproduction abnormality, including menstrual disorders, infertility and hyperandrogenism. Beyond hormonal derangements, obesity especially abdominal adipose accumulation, insulin resistance (IR), compensatory hyperinsulinemia, and a low-grade chronic inflammation often coexist with PCOS, which increase the risk for the development of metabolic syndrome, type 2 diabetes, and cardiovascular diseases in the future [2].

In recent years, dietary interventions in PCOS have become popular in both reproductive and endocrine researches. Since up to 60% of women with PCOS are overweight or obese [3], the International Evidence-based Guideline for the Assessment and Management of PCOS also emphasizes the importance of diet in PCOS and recommends dietary and exercise interventions as the first-line management in this population [4]. To date, several dietary strategies have been proposed for the treatment of PCOS, such as the low glycemic index diet, dietary approaches to stop hypertension diet, the Mediterranean diet, low carbohydrate diet, pulse-based diet, ketogenic diet, low-starch/low-dairy diet, and vegetarian diet [5–12]. Typically, intermittent fasting (IF) is the practice of alternate eating and fasting. IF is an umbrella term for three different types of diets: alternate-day fasting, the 5:2 diet, and time-restricted feeding (TRF) generally defined as fasting for 12–20 h [13]. TRF allows to ad libitum feeding within a large window of time each day without any calorie counting. Emerging evidence has suggested that TRF was beneficial for losing body weight, ameliorating IR, regulating metabolism, and improving cardiometabolic health [14, 15]. To the best of our knowledge, except for a very limited report on dawn-to-sunset Ramadan fasting in which Muslims abstain from eating and drinking [16], there has been no persuasive study on the possible role of IF in the PCOS population.

Since therapeutic options for PCOS are limited to oral contraceptives and metformin, and non-pharmacologic behavioral interventions such as TRF would be welcome additions to therapy for this common disorder. Thus a 6-week trial, with 2 consecutive periods: (1) 1-week baseline weight stabilization period; and (2) 5-week TRF period, was implemented to explore the effects of TRF on menstruation, gonadal and metabolic parameters in women with anovulatory PCOS and propose a basis for its inclusion in the treatment of PCOS.

## Materials and methods

### Participants

PCOS outpatients were recruited from the Department of Endocrinology, Shengjing Hospital of China Medical University in 2020 and data collection was completed in January 2021. The inclusion criteria were: age 18–40 years; body mass index (BMI) ≥ 24 kg/m^2^; anovulation; and a diagnosis of PCOS based on the Rotterdam diagnostic criteria [17]. The exclusion criteria were: use of medication therapy that impacts carbohydrate or lipid metabolism (oral contraceptive pills, insulin-sensitizers, anti-epileptics, anti-psychotics, statins, and fish oil) in the recent 6 months; body weight fluctuations for more than 5% in the past 3 months; in preparation for pregnancy, pregnant or lactating; perimenopausal; night-shift workers; fasting for more than 16 h per day; hypotension; patients with other diseases (such as congenital adrenal hyperplasia, Cushing syndrome, androgen-secreting tumors, hyperprolactinemia, diabetes, thyroid diseases, severe serious cardiovascular, gastrointestinal, kidney and liver diseases); alcohol intake for more than 100 g per week; smoking within the past 3 months and engaging in high-intensity exercise.

### Study design

The protocol for this study was approved by the Medical Research and New Technology Ethics Committee of Shengjing Hospital of China Medical University (reference: 2020PS682K). The study followed a pre-post non-randomized design and it was pre-registered on ClinicalTrials.gov (NCT04580433). The Strengthening the Reporting of Observational Studies in Epidemiology (STROBE) checklist for prospective cohort studies [18] was used to guide the report of this study (Additional file 1). The trial consisted of a 1-week baseline weight stabilization period followed by a 5-week TRF intervention period. After signing the informed consent, the following data were obtained to determine eligibility: (1) Height, weight, and age, (2) Blood pressure, (3) Menstrual cycle and (4) Medical history. The eligible participants were then invited to attend a baseline assessment visit where they completed the following assessments: (1) Body composition analysis and (2) Three Factor Eating Questionnaire Revised 21 Item (TFEQ-R21) questionnaire [19]. They also underwent a standard 75 g oral glucose tolerance test and blood samples for measurement of plasma glucose and insulin were drawn prior at 0, 60 and 120 min after glucose ingestion. During the baseline (week 0–1), participants were asked to measure their body weight every morning when fasting, continue with their usual diets, exercise, and sleep habits to keep their weight stable (no body weight fluctuations greater than 0.5 kg within a week). During the TRF period (week 2–6), they were asked not to change the composition of their usual diets but were instructed to eat freely from 8 am to 4 pm daily and to fast from 4 pm to 8 am the next day. Participants were provided with a food diary and were instructed to record their daily food intake from start to finish using the Boohee software, a diet and fitness app in China to calculate the corresponding calories. Daily dietary calorie intake was required to be approximately consistent with the baseline for as much as possible (fluctuations for no more than 10%) to address potential sources of bias. During the 16-h fasting, only water or calorie-free beverages were allowed, and participants were encouraged to drink enough water throughout the intervention period. We contacted participants via phone at the end of each week to review the protocol, monitor adverse events, provide support and guidance to promote compliance with interventions. All the baseline assessments were repeated at the follow-up visit on the 6th week when the diaries and data of time return to normal menstrual cycle were collected. All the examinations were conducted in the morning when the participants were fasting.

### Anthropometric measurements

For each participant, body weight and height were measured to calculate BMI [weight (kg) divided by height squared (m^2^), kg/m^2^]. Height was measured to the nearest 1 cm using a wall-mounted stadiometer (Seca 711; Hamburg, Germany). Body weight was determined to the nearest 0.1 kg using a multi-frequency bioelectrical impedance analyzer InBody 770 scanner (In-body Bldg, Seoul, Korea), with high resolution touch screen, frequency of 1,5, 50, 260, 500, 1000 kHz and measurement time of 60 s, with the subjects in a standing position with shoes, coats and sweaters removed, according to the manufacturer’s instructions. Waist-to-Hip Ratio (WHR) was measured by InBody 770 scanner, waist circumstance and hip circumstance were measured by the same nurse. The WHR was calculated using these measurements.

### Body composition

Body composition such as skeletal muscle mass (SMM) (kg), body fat mass (BFM) (kg), body fat percentage (BF%) and visceral fat area (VFA) (cm^2^) were evaluated by InBody 770 scanner.

### Blood sampling and analysis

The participants’ fasting blood samples were collected before and after the intervention. Blood samples were taken from antecubital vein and collected into the BD Vacutainers Tubes (SSTTM II Advance, REF 367953). Then, samples were centrifuged for 10 min at 3600 rpm at 4 °C. The obtained serum and plasma were aliquoted and stored at – 80 °C before analysis. Insulin 0 min, 60 min, 120 min (μU/mL) were measured by radioimmunological assay, luteinizing hormone (LH) (mIU/mL) and follicle-stimulating hormone (FSH) (mIU/mL) were measured by chemiluminescent immunoassay, total testosterone (TT) (ng/mL) was measured by electrochemiluminescent immunoassay, insulin-like growth factor 1 (IGF-1) (ng/mL) and sex hormone-binding globulin (SHBG) (nmol/L) were measured by immunochemiluminescent on Beckman Coulter Unicel Dxl 800. Total cholesterol (TC) (mmol/L) was measured by cholesterol oxidase method, triglycerides (TG) (mmol/L) was measured by deionization & enzyme method, and low density lipoprotein-cholesterol (LDL-C) (mmol/L) was measured by selective solubilization method, alanine aminotransferase (ALT) (U/L) and aspartate aminotransferase (AST) (U/L) were measured by NADH method, uric acid (UA) (umol/L), glucose 0 min, 60 min, 120 min (mmol/L) and high-sensitivity C-reactive protein (hs-CRP) (mg/L) were measured separately by uricase-PAP method, hexokinase method and rate nephelometry method on ci 16200 Abbott Architect analyzer. Homeostasis model assessment-insulin resistance (HOMA-IR) was calculated according to the formula “FINS (μU/mL) × FG (mmol/L)/22.5”. Free androgen index (FAI) (%) was calculated according to the formula “TT (ng/mL) × 100/SHBG (nmol/L)”.

### Questionnaires

The TFEQ-R21 were completed during pre- and post- visits. The TFEQ-R21 covers 3 eating behavior domains: the cognitive restraint scale (6 items) assesses control over food intake to influence body weight and body shape; the emotional eating scale (6 items) measures the propensity to overeating in relation to negative mood states; the uncontrolled eating scale (9 items) assesses the tendency to lose control of overeating when feeling hungry or when exposed to external stimuli. It contains 20 questions based on a 4-point Likert-Scale (1 = definitely false, 2 = mostly false, 3 = mostly true, 4 = definitely true) and each question scored between 1 and 8 on eating restraint (1 = no restraint when eating, 8 = extreme restraint when eating). Higher scores indicate greater cognitive restraint, uncontrolled eating, or emotional eating.

### Statistical analysis

The distribution of continuous data was tested with the Anderson–Darling test. Continuous variables were presented as mean ± standard deviation (mean ± SD) (normally distributed) or median (25th–75th percentile) (non-normally distributed) form and were analyzed using Student’s test for matched pairs (normally distributed) or Wilcoxon matched pairs signed-rank test (non-normally distributed) to compare parameters before and after 5 weeks of the TRF period. All data analyses were performed in GraphPad Prism (version 8.0.1; GraphPad Software). Significance was considered when *P* < 0.05. For responses with missing values, the values were not included in the analyses.

## Results

### Participants

**Fig. 1:**
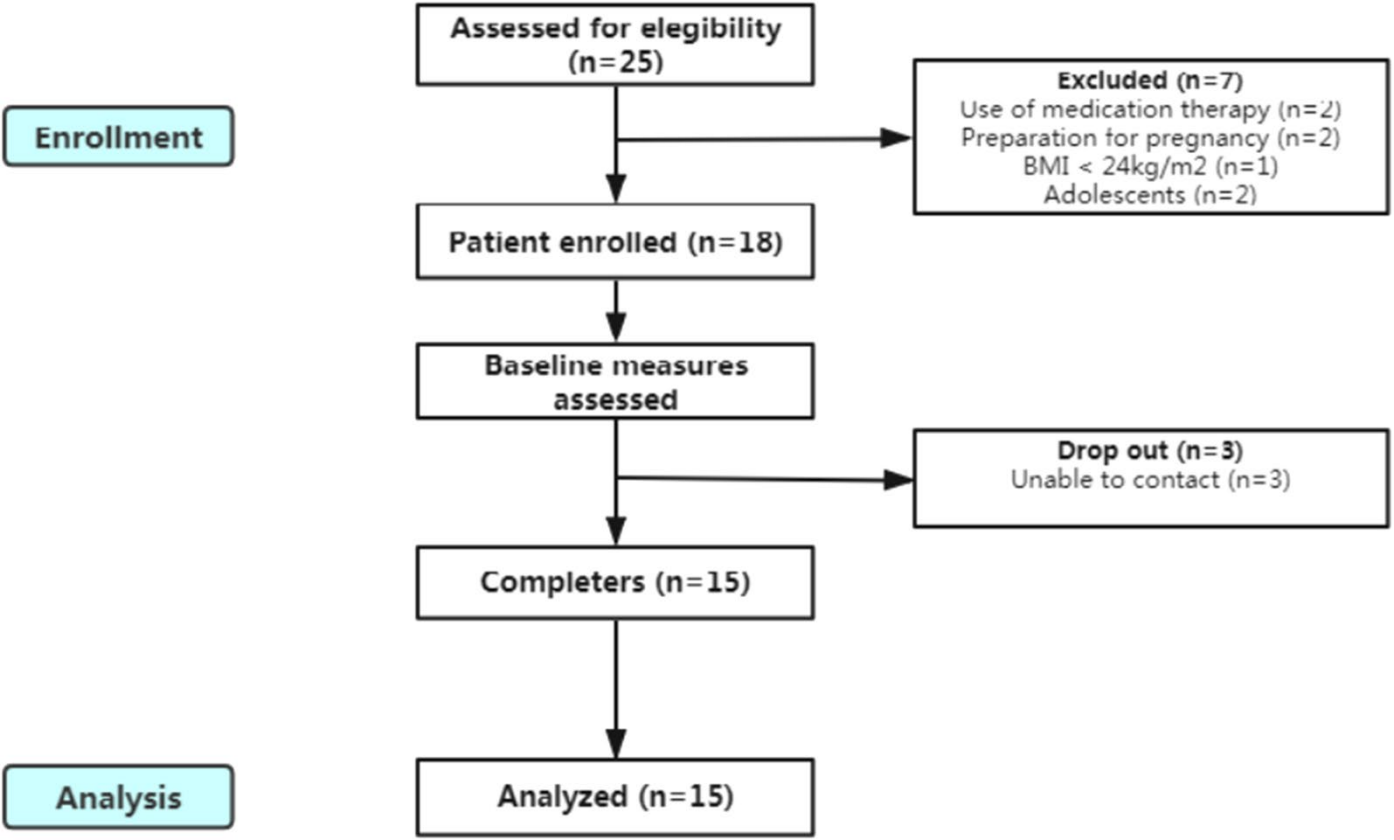
Flow chart from the study design

Twenty five participants were recruited to participate in the investigation, seven women were excluded: two for current PCOS pharmacological therapy, two for preparation for pregnancy, one for BMI < 24 kg/m^2^ and two were adolescents. Only fifteen participants completed the 6-week of follow-up with three dropouts could not be contacted at the end of the first week. Thus fifteen subjects were concluded in the study with 31 variables of interests (Fig. 1). Participants were between 18 and 31 years old with anovulation (the menstrual cycle was delayed by 3 months–3 years) and normotensive but mainly insulin resistant (patients at the beginning of the study had a HOMA-IR higher than 2.3). All PCOS participants were diagnosed with hyperandrogenemia within 1 month. Three participants caught a cold during the follow-up and the values of hsCRP of them were excluded, but by the time of the blood collection, the treatments were already completed, and the participants were free of symptoms. One participant was not in fasting state at the follow-up, so we did not test her blood for glucose and insulin analysis. Feeling hungry every day was reported by two participants (2/15, 13.3%), several days per week by three participants (3/15, 20.0%), once a week or less by ten participants (7/15, 46.7%), and never by three participants (3/15, 20.0%). They did not have any discomfort, except for one participant complained that she had irregular defecation in the first week of TRF. Besides, TRF did not influence their sleep. Seven participants (7/15, 46.7%) found it easy or very easy to adhere to TRF rules, six participants (6/15, 40.0%) found it neither easy nor difficult, while two participants (2/15, 13.3%) found it difficult or very difficult. All participants said that they wanted to continue and four participants (4/15, 26.7%) said that they would recommend TRF to others.

### Anthropometric and body composition measurements

Subjects lost a modest amount of average 1.3 kg weight (1.7% of their body weight) from baseline. The study also revealed significant reductions in BMI (*P* < 0.001), BFM (*P* < 0.001), BF% (*P* = 0.012) and VFA (*P* = 0.015). There were no significant differences in WHR and SMM. The results are presented in Table 1.

**Table 1:** Anthropometric measurements and body composition

	Pre	Post	*P*-value
Body weight (kg)	74.70 (69.80–97.50)	73.40 (68.40–95.50)	< 0.001*
BMI (kg/m^2^)	29.75 ± 4.31	28.57 ± 4.41	< 0.001*
WHR	0.93 ± 0.05	0.92 ± 0.05	0.050
SMM (kg)	25.41 ± 3.96	24.77 ± 3.98	0.062
BFM (kg)	35.28 ± 10.03	32.89 ± 9.91	< 0.001*
BF%	40.65 (39.83–47.63)	39.65 (38.38–45.98)	0.012*
VFA (cm^2^)	164.8 ± 39.45	154.7 ± 41.42	0.015*

BMI: Body mass index; WHR: waist-to-Hip Ratio; SMM: Skeletal muscle mass; BFM: body fat mass; BF%: body fat percentage; VFA: visceral fat area

The comparison across timepoints has been assessed using Paired t test and Wilcoxon test

Results are expressed as mean ± SD or median (25th–75th percentile)

^*^P < 0.05

### Metabolic parameters

**Fig. 2:**
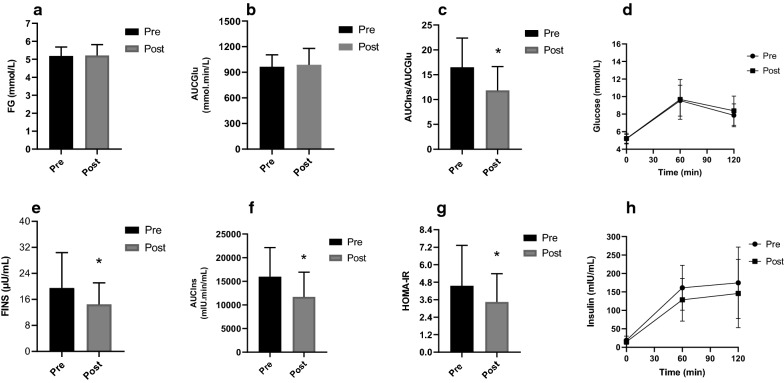
Changes in glucose and insulin metabolism variables after TRF period. FG: fasting glucose; FINS: fasting insulin; HOMA-IR: homeostasis model assessment-insulin resistance; AUCIns: area under the curve (AUC) for insulin; AUCGlu: area under the curve (AUC) for glucose. Standard deviation is represented in the figure. *P < 0.05

Multivariate analyses of insulin resistance (both AUCIns/AUCGlu and HOMA-IR) were performed. Significant decreases were observed in FINS (*P* = 0.017), HOMA-IR (*P* = 0.025), AUCIns (*P* = 0.007), AUCIns/AUCGlu (*P* = 0.001), while there were no significant changes in FG, AUCGlu, TG, TC and LDL-C (Fig. 2). The data are reported in Table 2.

**Table 2:** Metabolic parameters

	Pre	Post	*P*-value
FG (mmol/L)	5.08 (4.76–5.60)	4.97 (4.76–5.66)	0.614
FINS (μU/mL)	15.60 (13.45–25.00)	12.30 (10.30–17.30)	0.017*
AUCIns (mU/L*min)	15,974 ± 6158	11,694 ± 5230	0.007*
AUCGlu (mmol/L*min)	963.8 ± 140.9	988.3 ± 190.7	0.516
AUCIns/AUCGlu	16.49 ± 5.90	11.84 ± 4.80	0.001*
HOMA-IR	3.45 (2.91–5.64)	2.73 (2.27–3.85)	0.025*
TG (mmol/L)	1.23 (0.94–1.68)	1.05 (0.70–1.67)	0.715
TC (mmol/L)	4.57 ± 0.75	4.43 ± 0.66	0.328
LDL-C (mmol/L)	2.76 ± 0.61	2.75 ± 0.62	0.984

FG: Fasting glucose; FINS: fasting insulin; AUCIns: area under the curve (AUC) for insulin; AUCGlu: area under the curve (AUC) for glucose; HOMA-IR: homeostasis model assessment-insulin resistance; TG: triglycerides; TC: total cholesterol; LDL-C: low density lipoprotein-cholesterol

The comparison across timepoints has been assessed using Paired t test and Wilcoxon test

Results are expressed as mean ± SD or median (25th–75th percentile)

^*^P < 0.05

### Menstruation and gonadal parameters

**Fig. 3:**
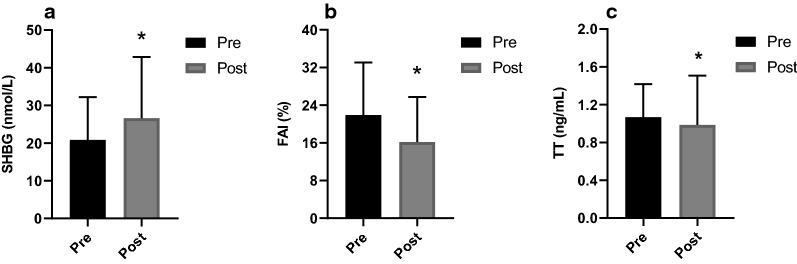
Changes in androgen-related variables after TRF period. TT: total testosterone; SHBG: sex hormone-binding globulin; FAI: free androgen index. Standard deviation is represented in the figure. *P < 0.05.

At the end of the study, an improvement in menstrual cycle irregularity was detected in 73.3% (11/15) participants. For gonadal parameters, there were a significant increase in SHBG (*P* < 0.001) and decrease in TT (*P* = 0.048) and FAI (*P* = 0.001) (Fig. 3). There were no significant changes in LH, FSH and LH/FSH. The data are reported in Table 3.

**Table 3:** Gonadal parameters

	Pre	Post	*P*-value
TT (ng/mL)	1.00 (0.77–1.26)	0.91 (0.61–1.09)	0.048*
SHBG (nmol/L)	19.00 (11.70–25.10)	22.70 (15.20–33.90)	< 0.001*
FAI (%)	21.91 ± 11.17	16.20 ± 9.56	0.001*
LH (mIU/mL)	13.09 ± 4.83	10.67 ± 5.22	0.176
FSH (mIU/mL)	5.66 (5.06–7.09)	5.21 (4.32–5.51)	0.252
LH/FSH	2.21 (1.72–2.52)	1.81 (1.43–3.07)	0.515

TT: Total testosterone; SHBG: sex hormone-binding globulin; FAI: free androgen index; LH: luteinizing hormone; FSH: follicle-stimulating hormone

The comparison across timepoints has been assessed using Paired t test and Wilcoxon test

Results are expressed as mean ± SD or median (25th–75th percentile)

^*^P < 0.05

### Eating behaviors

According to TFEQ-R21, cognitive restraint, uncontrolled eating and emotional eating did not change over TRF period. The data are reported in Table 4.

**Table 4:** Eating behaviors

	Pre	Post	*P*-value
Cognitive restraint	15.00 ± 4.29	17.80 ± 2.62	0.067
Emotional eating	13.13 ± 5.19	12.20 ± 3.78	0.332
Uncontrolled eating	19.13 ± 4.16	18.67 ± 2.44	0.669

The comparison across timepoints has been assessed using paired t test

Results are expressed as mean ± SD

^*^P < 0.05

### Other parameters

Compared to baseline, there were a significant decrease in hsCRP (*P* = 0.040) and ALT levels (*P* = 0.027) and a significant rise in IGF-1 level (*P* = 0.006). There were no significant changes in UA and AST levels. The data are reported in Table 5.

**Table 5:** Other parameters

	Pre	Post	*P*-value
hs-CRP (mg/L)	4.91 ± 3.21	2.86 ± 1.55	0.040*
IGF-1 (ng/mL)	157.5 ± 31.94	210.3 ± 51.84	0.006*
UA (umol/L)	418.2 ± 76.75	407.7 ± 122.6	0.656
AST (U/L)	25.50 (15.75–38.00)	18.00 (16.00–31.00)	0.113
ALT (U/L)	47.67 ± 37.95	32.58 ± 26.11	0.027*

hs-CRP: High-sensitivity C-reactive protein; IGF-1: insulin-like growth factor 1; ALT: alanine aminotransferase; AST: aspartate aminotransferase; UA: uric acid

The comparison across timepoints has been assessed using Paired t test and Wilcoxon test

Results are expressed as mean ± SD or median (25th–75th percentile)

^*^P < 0.05

## Discussion

This is the first study to investigate the implications of TRF in PCOS patients with chronic anovulation on anthropometric indexes, body composition, endocrine and metabolic profiles. Five weeks of TRF improved menstruation, gonadal profiles (TT, SHBG and FAI), body weight, BMI, body composition profiles (BFM, BF% and VFA), hyperinsulinemia and insulin resistance profiles (FINS, HOMA-IR, AUCIns and AUCIns/AUCGlu), decreasing ALT, hsCRP and increasing IGF-1 in a group of fifteen young women with anovulatory PCOS.

Hyperandrogenemia is one of the main features of PCOS which often leads to irregular menstruation. In the present study, all the patients were anovulatory women with diagnosed or previously diagnosed hyperandrogenemia. After TRF, more than half of the patients restored their normal menstrual cycles. Hence, it is promising that TRF may ameliorate hyperandrogenemia by increasing SHBG levels and exert beneficial effects on the recovery of the normal menstrual cycle in PCOS patients. Abnormal LH/FSH ratio is common in women with PCOS. A prior study had shown that TRF had a negative effect on LH pulsation during ovarian development in prepubertal girls [20], whereas we did not find any significant difference in LH, FSH and LH/FSH. Paoli et al. [12] investigated the effect of a ketogenic diet on PCOS patients and observed significant decreases in TT, free testosterone, LH/FSH ratio and LH, with a significant reduction of body weight (− 9.43 kg) after twelve weeks treatment. However, whether the benefits were from weight loss or from the specific dietary approach still needed to be investigated. Furthermore, the duration of our study was short (5 weeks) which might limit the possible changes in LH and FSH levels.

Obesity is closely related to PCOS. A higher BMI is associated with a greater prevalence of menstrual irregularity, hyperandrogenemia and hirsutism [21]. Accumulating evidence shows that TRF may produce a small but statistically significant 1–4% of weight loss [22]. Previous meta-analyzes have also shown that TRF was more likely to control weight and improve body composition [13, 23]. For active females, resistant training combined with TRF may result in a greater loss of fat mass than resistant training with a usual diet, and TRF group also reported a 18% decrease in visceral fat mass [24]. In our study, participants who maintained dietary intake of past habits with a low intensity of exercise also reduced their body weight, BMI, BFM, BF% and VFA significantly in the TRF period, while TRF for 5 weeks did not decrease WHR or change lean mass significantly.

Elevated blood glucose concentration is a pivotal factor in the diagnosis of metabolic diseases. Consistently high concentration of glucose can damage blood vessels and lead to an increased heart disease risk and insulin resistance [22]. While previous findings on the role of TRF in fasting glucose were equivocal, most of these trials reported no change in fasting glucose [22], it is worth noting that although Martens et al. [24] reported no change in fasting glucose, they did find a significant decrease in AUCGlu during the 8 h TRF. A five-day TRF (10 am–5 pm) reduced night-time glucose level in participants who were overweight [25]. A 4-day TRF (8 am–2 pm) also lowered the mean glucose level at night-time but did not lower glucose level during the awake period. In summarizing the results of the entire day, TRF reduced mean 24 h glucose levels by 4 ± 1 mg/dL [26]. In our study, blood tests were all conducted in the morning and we did not detect significant differences in FG and AUCGlu. A review reported that isocaloric TRF seemed to be more beneficial in reducing FINS and IR when compared to ad libitum TRF [22]. Isocaloric TRF may improve fasting insulin levels independently of weight loss, especially in those who are prediabetes [27]. Our ad libitum study observed significant reductions in FINS, HOMA-IR, AUCIns and AUCIns/AUCGlu in a short time TRF (8 am–4 pm) which suggested that TRF without limiting energy may also ameliorate hyperinsulinemia and improve IR.

Dyslipidemia is present in 70% of patients with PCOS, regardless of BMI [28]. The roles of TRF in plasma lipid levels were not consistent. No significant differences were found in TC, TG and LDL-C levels in the present study.

Polycystic ovaries show persistent chronic inflammation with a larger number of infiltrating inflammatory cells. These cells induce insulin resistance, stimulate androgen production and disrupt the function of hypothalamic-pituitary-ovarian axis. A meta-analysis showed that CRP level was higher in PCOS patients than in healthy women, independent of obesity [29]. We found that short time TRF may reduce hsCRP and thus improve the state of chronic inflammation in overweight PCOS patients. IGF-1 plays an important role in glucose metabolism [30]. Low circulating levels of IGF-1 in healthy adults are associated with reduced β-cells function [31]. The increased level of IGF-1 after the reduction diet had a cardioprotective effect [32]. We also found a higher IGF-1 in PCOS patients after TRF, which was contrary to the results of a 5-day TRF in overweight humans [26]. While it is still difficult to clarify the effect of IGF-1 in PCOS, since IGF-1 has linked with the disturbed follicular development in PCOS [33]; a higher IGF-1 in PCOS women may be related to the increased vascularity that underlies the increased blood flow [34]. Abnormal serum ALT is associated with impaired insulin sensitivity in young women with PCOS in a manner that is independent from the contribution of age and total adiposity [35]. Our study found TRF may decrease ALT levels in PCOS.

There were several limitations in the current study. Firstly, this was a non-randomized intervention without a control group with a small number of participants which limited our ability to detect a significant difference. Secondly, our study was of a short duration (5 weeks) which limited the physiological changes that could be induced. Because of the strict inclusion criteria, the variability of the cohort may have limited the generalizability of the results to a broader cohort of patients with PCOS. Thirdly, the intervention was conducted in a free-living population, and participants failed to receive standardized diets. Although the participants were asked to record calories to retain their energy intake from food, the estimated value is for reference only. Fourthly, adherence to the 8 h TRF may have been affected by various factors since participants may be more likely to adhere if they feel in control. Thus, well-designed studies are needed to examine the safety, applicability, and usefulness of TRF for PCOS patients.

## Conclusions

Eight-hour TRF may have beneficial effects on improving menstruation, hyperandrogenemia and reducing weight especially body fat, decreasing insulin resistance and chronic inflammation in women with anovulatory PCOS. TRF may be suitable for PCOS women with appropriate counseling and patient management.

## Supplementary Information

**Additional file 1. **STROBE Statement.

## Data Availability

The datasets used and/or analysed during the current study are available from the corresponding author on reasonable request.

## Abbreviations

PCOS: Polycystic ovary syndrome
IF: Intermittent fasting
TRF: Time-restricted feeding
TFEQ-R21: Three Factor Eating Questionnaire Revised 21 Item
BMI: Body mass index
WHR: Waist-to-Hip Ratio
SMM: Skeletal muscle mass
BFM: Body fat mass
BF%: Body fat percentage
VFA: Visceral fat area
FG: Fasting glucose
FINS: Fasting insulin
IR: Insulin-resistance
HOMA-IR: Homeostasis model assessment-insulin resistance
AUCIns: Area under the curve for insulin
AUCGlu: Area under the curve for glucose
LDL-C: Low density lipoprotein-cholesterol
TC: Total cholesterol
TG: Triglycerides
TT: Total testosterone
SHBG: Sex hormone-binding globulin
FAI: Free androgen index
LH: Luteinizing hormone
FSH: Follicle-stimulating hormone
hs-CRP: High-sensitivity C-reactive protein
IGF-1: Insulin-like growth factor 1
ALT: Alanine aminotransferase
AST: Aspartate aminotransferase
UA: Uric acid
SD: Standard deviation
STROBE: The Strengthening the Reporting of Observational Studies in Epidemiology
